# Blood biomarkers of response to concomitant intratympanic and systemic corticosteroids treatment for idiopathic sudden sensorineural hearing loss

**DOI:** 10.1007/s00405-025-09904-w

**Published:** 2025-12-22

**Authors:** Giancarlo Tirelli, Ludovica Costariol, Federico Cavallo-Ronchi, Egidio Sia, Simone Zucchini, Paolo Boscolo-Rizzo

**Affiliations:** Department of Medical, Surgical and Health Sciences, Section of Otolaryngology, University of Trieste, Strada di Fiume 447, Trieste, 34149 Italy

**Keywords:** Idiopathic sudden sensorineural hearing loss, Corticosteroid therapy, Intratympanic injection, Inflammatory biomarkers, Platelet-to-lymphocyte ratio, Hearing recovery

## Abstract

**Purpose:**

To evaluate the prognostic value of hematological inflammatory biomarkers in predicting hearing recovery in patients with idiopathic sudden sensorineural hearing loss (ISSNHL) treated with combined systemic and intratympanic corticosteroids.

**Methods:**

This retrospective study included 125 patients diagnosed with ISSNHL between 2012 and 2021 who received both oral prednisone and intratympanic methylprednisolone. Clinical data and pre-treatment blood samples were collected. Inflammatory indices including neutrophil-to-lymphocyte ratio (NLR), platelet-to-lymphocyte ratio (PLR), lymphocyte-to-monocyte ratio (LMR), systemic immune-inflammation index (SII), and systemic inflammation response index (SIRI) were calculated. Hearing outcomes were classified as complete, partial, or no recovery based on pure tone average (PTA) thresholds. Univariate and multivariable logistic regression analyses were performed to identify predictors of recovery.

**Results:**

Of the 125 patients, 33 (26.4%) had no recovery, 50 (40.0%) partial recovery, and 42 (33.6%) complete recovery. Dizziness, current smoking, delayed treatment initiation (> 7 days), elevated triglycerides (≥ 98 mg/dL), and high PLR (≥ 142) were independently associated with a lower probability of hearing recovery. A higher LMR (≥ 3.0) showed a positive trend but did not reach statistical significance in adjusted models.

**Conclusions:**

In patients with ISSNHL treated with dual-route corticosteroids, several clinical and hematologic factors, particularly elevated PLR and delayed treatment, are independently associated with reduced odds of hearing recovery. Inflammatory blood markers may serve as accessible prognostic tools to support early risk stratification.

## Introduction

Sudden sensorineural hearing loss (SSNHL), defined by a rapid decrease in hearing of 30dB Hearing Level (HL) or more across at least three contiguous frequencies [1], constitutes a significant global health issue, with an estimated annual incidence rate between 5 and 27 cases per 100,000 inhabitants [2]. Despite its potential for spontaneous recovery sometimes masking its prevalence, this condition may result in persistent hearing loss and tinnitus thus highly impacting on patient quality of life [3]. SSNHL is predominantly unilateral with the prevalence of bilateral cases varying from 0.4 to 3.4%. Approximately 90% of SSNHL cases present without an identifiable cause, with various etiologies such as viral infections, vascular anomalies, coagulation disorders, or chronic inflammation being hypothesized [4].

The management and recovery prospects of SSNHL are highly variable, reflecting its complexity and multifactorial nature [5]. While adopted treatments range from absolute rest, carbogen inhalation, use of anti-inflammatories, vasodilators, antivirals, anticoagulants, to hyperbaric oxygen therapy, corticosteroids remain the cornerstone of therapeutic strategies, aimed at reducing inflammation in the cochlea and auditory nerve [5].

Several studies in the field of oncology have highlighted the importance of various markers and inflammatory blood indices that would reflect the local tumor inflammatory microenvironment and have been shown to predict prognosis in patients with different types of neoplasms [6, 7]. Therefore, systemic immune responses may mirror localized pathological events whose characteristics could influence the natural history of the disease [8]. A similar hypothesis has been formulated in the context of SSNHL. Previous studies have already investigated the efficacy of certain hematological biomarkers in predicting auditory recovery in patients with SSNHL [9, 10].

The primary objective of this study was to examine different inflammatory hematological biomarkers, thus expanding the scope of previous studies, to assess their capacity in predicting auditory recovery in patients with idiopathic SSNHL (ISSNHL) treated with concomitant intratympanic and systemic corticosteroids administration.

## Materials and methods

### Study subjects

This retrospective observational study was conducted at the Otorhinolaryngology Clinic of the University of Trieste, Italy. We reviewed the clinical records of consecutive patients diagnosed with ISSNHL and treated between September 2012 and December 2021 with a combined regimen of systemic and intratympanic corticosteroids. Inclusion criteria were: age ≥ 18 years; unilateral sensorineural hearing loss of at least 30 dB HL affecting three or more consecutive frequencies, with onset within 72 h; time from symptom onset to treatment ≤ 6 weeks; no prior therapy for ISSNHL before admission; availability of haematological parameters measured before initiation of treatment, including complete blood count with differential (neutrophils, lymphocytes, monocytes), platelets, glucose, triglycerides, total cholesterol, HDL, LDL, and C-reactive protein (CRP); tonal audiometry performed before and after treatment; and MRI imaging to exclude retrocochlear or central causes. Exclusion criteria were: conductive or mixed hearing loss; bilateral involvement; Ménière’s disease; retrocochlear lesions; cranial trauma; malformations of the middle or inner ear; ototoxic drug exposure; inflammatory middle ear conditions; and any previous use of corticosteroids. Patients with missing laboratory or audiometric data were also excluded.

### Laboratory parameters and derived indices

Laboratory data collected at admission included haemoglobin, red and white blood cell counts, neutrophils, lymphocytes, monocytes, platelets, glucose, triglycerides, cholesterol (total, HDL, LDL), and CRP. Inflammatory indices were calculated as follows: Neutrophil-to-lymphocyte ratio (NLR); Platelet-to-lymphocyte ratio (PLR); Lymphocyte-to-monocyte ratio (LMR); Systemic immune-inflammation index (SII = platelets × neutrophils/lymphocytes); Systemic inflammation response index (SIRI = neutrophils × monocytes/lymphocytes).

### Treatment

The treatment protocol included both intratympanic and systemic corticosteroids. Intratympanic methylprednisolone (125 mg/2 mL) was administered once daily for three consecutive days under otomicroscopy, with a volume of 0.5 to 1.0 mL per infiltration. At the same time, patients began oral prednisone at a daily dose of 60 mg for 14 days, followed by tapering by 10 mg per day over the next five days.

### Post-Treatment evaluation

A tonal audiometry was performed in a soundproof booth 30 days after the completion of treatment, and the treatment efficacy was evaluated. We classified hearing recovery as follows: “Complete recovery” was determined when the PTA at frequencies from 500 Hz to 4 kHz returned to within 10 dB HL of the unaffected ear. “No recovery” was defined as less than 10 dB HL improvement in the PTA. In all other scenarios, recovery was classified as “partial recovery”.

### Statistical analysis

Descriptive statistics were used to summarize socio-demographic, clinical, and laboratory characteristics of the study population. Continuous variables were expressed as median and interquartile range (IQR) and compared across treatment outcome groups (no recovery, partial recovery, complete recovery) using non-parametric analysis of variance for trend. Categorical variables were reported as frequencies and percentages and compared using Fisher’s exact test. To assess the association between hematological and clinical variables and a 3-leve treatment response (i.e., no recovery, partial recovery, and total recovery), univariate and multivariable multinomial logistic regression analyses were performed. Odds ratios (ORs) and corresponding 95% confidence intervals (CIs) were calculated to estimate the strength of associations between potential predictors and hearing recovery outcomes, both for partial/complete recovery versus no recovery and for complete recovery versus partial/no recovery. For the multivariable models, variables were selected based on clinical relevance and results from univariate analysis. The alpha level for statistical significance was set at 0.05, and statistical analysis was carried out using R software (version 4.2.3, 2023).

## Results

A total of 125 patients were included in the analysis and categorized based on treatment outcomes: no recovery (*n* = 33), partial recovery (*n* = 50), and complete recovery (*n* = 42).

Among clinical and demographic characteristics (Table 1), dizziness was significantly more prevalent in the no recovery group compared to the other two groups (*p* < 0.001). No significant differences were observed for age, sex, smoking status, or presence of tinnitus. The time from symptom onset to treatment initiation was significantly longer in patients without recovery (*p* < 0.001) (Table 2).

**Table 1:** Socio-demographic and clinical characteristics according to treatment outcome

	No recovery (*n* = 33)		Partial recovery (*n* = 50)		Complete recovery (*n* = 42)		Fisher’s exact test (*p*)
Age (years)	62.4 (50.9–74.6)		64.7 (52.4–70.3)		55.7 (44.4–68.0)		0.155^a^
Sex							
Man	22	(31.4)	22	(31.4)	26	(37.1)	0.089
Woman	11	(20.0)	28	(50.9)	16	(29.1)	
Tobacco smoking							
Never	16	(24.2)	24	(36.4)	26	(39.4)	0.196
Former	5	(17.2)	14	(48.3)	10	(34.5)	
Current	12	(40.0)	12	(40.0)	6	(20.0)	
Diabetes mellitus							
No	27	(26.7)	40	(39.6)	34	(33.7)	1.000
Yes	6	(25.0)	10	(41.7)	8	(33.3)	
Tinnitus							
No	12	(36.4)	8	(24.2)	13	(39.4)	0.080
Yes	21	(22.8)	42	(45.7)	29	(31.5)	
Dizziness							
No	19	(20.2)	35	(37.2)	40	(42.6)	< 0.001
Yes	14	(45.2)	15	(48.4)	2	(6.5)	

^a^p for trend estimated through a non-parametric analysis of variance

**Table 2:** Blood parameters and inflammatory blood markers according to treatment outcome

	No recovery (*n* = 33)	Partial recovery (*n* = 50)	Complete recovery (*n* = 42)	*p*_trend_^a^
PTA loss	45 (31–69)	54 (41–78)	35 (30–63)	0.325
Time to treatment (days)	14.0 (8.0–30.0)	5.5 (3.0–21.0)	6.0 (4.0–12.0)	< 0.001
Platelet count (x10^3^/mL)	215 (194–262)	238 (183–265)	219 (205–270)	0.774
Haemoglobin (g/dL)	14.0 (13.3–14.9)	13.9 (13.2–14.8)	14.2 (13.3–15.1)	0.972
Neutrophils (x10^3^/mL)	5.16 (3.76–7.13)	5.26 (3.97–6.71)	5.15 (3.54–7.33)	0.894
Lymphocytes (x10^3^/mL)	1.27 (1.01–1.85)	1.73 (1.32–2.15)	5.15 (3.54–7.33)	0.014
Monocytes (x10^3^/mL)	0.57 (0.38–0.74)	0.54 (0.41–0.68)	0.55 (0.46–0.65)	0.962
Glucose (mg/dL)	101 (96–113)	101 (94–124)	102 (94–120)	0.747
Triglycerides (mg/dL)	205 (180–247)	84 (70–109)	96 (80–115)	0.011
Cholesterol (mg/dL)	205 (180–247)	187 (150–210)	176 (145–223)	0.033
HLD-cholesterol (mg/dL)	55 (45–65)	58 (49–67)	55 (48–65)	0.663
C-reactive protein	1.0 (0.6–2.1)	1.6 (0.9–2.3)	1.4 (1.0–3.1.0.1)	0.078
NLR	3.96 (2.52–7.03)	3.13 (2.25–5.14)	2.86 (1.93–4.91)	0.099
PLR	186.1 (118.1–227.7.1.7)	134.7 (103.6–166.2.6.2)	122.8 (93.6–192.4.6.4)	0.027
LMR	2.70 (2.10–3.34)	3.15 (2.54–3.93)	3.32 (2.58–4.04)	0.017
SII	816 (610–1685)	716 (377–1218)	682 (414–1208)	0.139
SIM	2.04 (1.23–2.79)	1.60 (1.04–2.71)	1.58 (1.05–2.44)	0.161

^a^Estimated through a non-parametric analysis of variance

Several inflammatory and haematological markers showed significant associations with treatment outcome. Triglyceride and cholesterol levels were higher in the no recovery group (*p* = 0.011 and *p* = 0.033, respectively). The PLR was elevated in the no recovery group, while the LMR was lower compared to the other groups (*p* = 0.027 and *p* = 0.017, respectively) (Table 2).

**Fig. 1:**
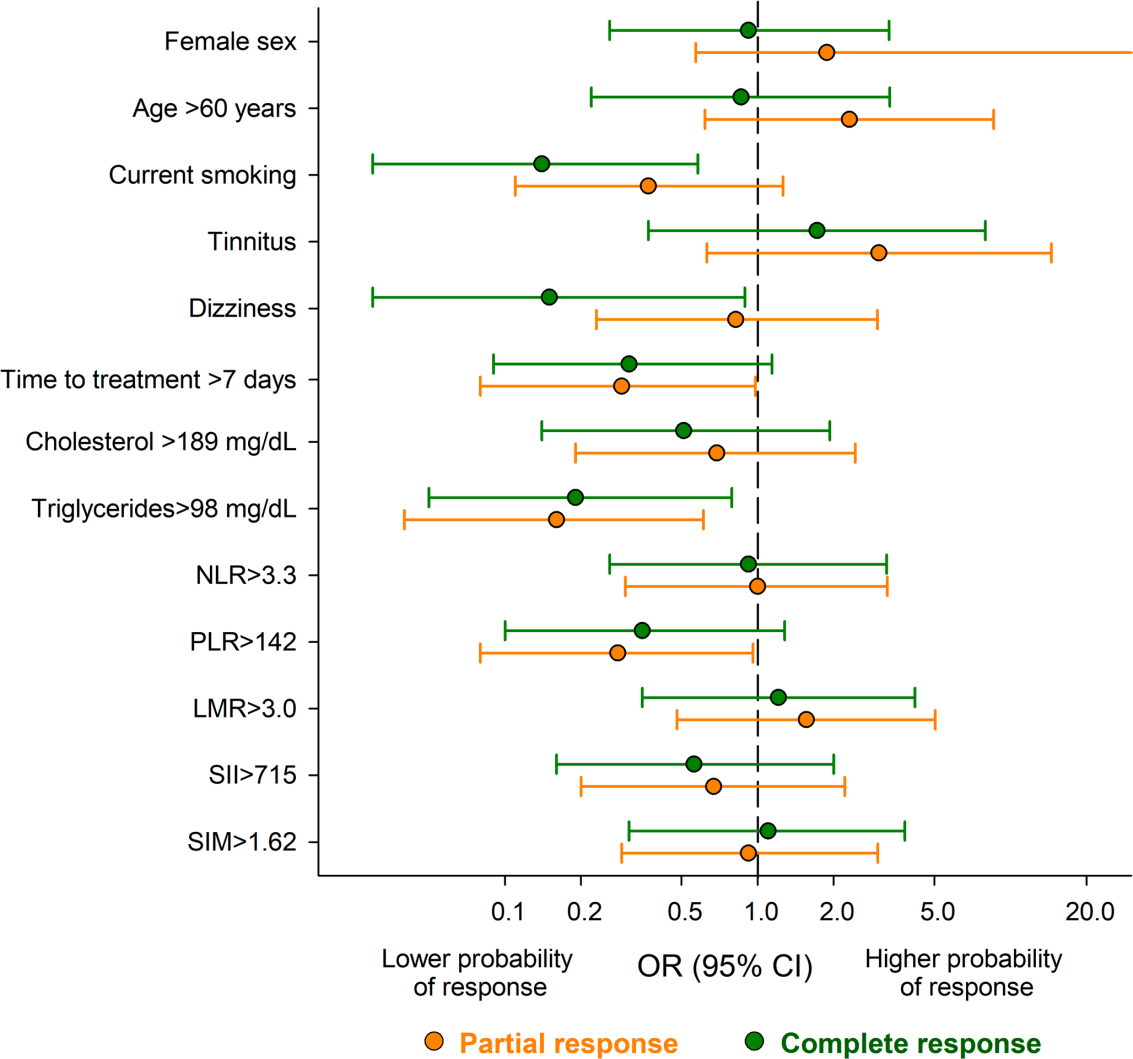
Odds ratio (OR) and corresponding 95% confidence intervals (CI) for partial and complete response according to sociodemographic and clinical characteristics

In multivariable logistic regression analysis, current smoking, presence of dizziness, delayed treatment (> 7 days), elevated triglycerides (≥ 98 mg/dL), and high PLR (≥ 142) were independently associated with a lower likelihood of hearing recovery (p-values ranging from 0.003 to 0.030). A higher LMR (≥ 3.0) was associated with better outcome in univariate analysis, though it did not retain significance in the adjusted model (Table 3). Adjusted ORs and 95% confidence intervals for the main predictors of hearing recovery are presented visually in Fig. 1. Notably, dizziness, smoking, treatment delay, high triglycerides, and PLR were all associated with a reduced probability of recovery.

**Table 3:** Odds ratio (OR) and corresponding 95% confidence interval (CI)^a^ for partial and total recovery versus no recovery, according to socio-demographic characteristics, clinical features and blood markers

	OR (95% CI)		OR (95% CI)^b^	
	Partial	Total	Partial	Total
Female sex	2.55 (1.02–6.35)	1.23 (0.47–3.20)	1.88 (0.57–6.23)	0.92 (0.26–3.31)
Age ≥ 60 years	1.30 (0.58–3.15)	0.58 (0.23–1.48)	2.31 (0.62–8.59)	0.86 (0.22–3.33)
Current smoking	0.55 (0.21–1.45)	0.29 (0.10–0.89)	0.37 (0.11–1.26)	0.14 (0.03–0.58)
Diabetes mellitus	1.13 (0.37–3.46)	1.60 (0.33–3.42)	2.13 (0.47–9.73)	1.60 (0.30–8.55)
Tinnitus	3.00 (1.06–8.46)	1.28 (0.49–3.35.49.35)	3.02 (0.63–14.5)	1.72 (0.37–7.97)
Dizziness	0.58 (0.23–1.46)	0.07 (0.01–0.33)	0.82 (0.23–2.98)	0.15 (0.03–0.89)
PTA loss ≥ 47.5	1.80 (0.74–4.38)	0.90 (0.36–2.25)	1.46 (0.45–4.77)	1.01 (0.29–3.52)
Time to treatment > 7 days	0.30 (0.11–0.78)	0.22-0.08.22.08-0.60()	0.29 (0.08–0.98)	0.31 (0.09–1.14)
Platelets ≥ 225 × 10^3^/mL	1.66 (0.68–4.02)	0.99 (0.40–2.48)	1.34 (0.41–4.37)	0.85 (0.24–2.99)
Neutrophils ≥ 5.25 × 10^3^/mL	1.25 (0.52–3.01)	0.97 (0.39–2.41)	1.32 (0.39–4.44)	1.00 (0.28–3.59)
Lymphocytes ≥ 1.65 × 10^3^/mL	2.23 (0.90–5.49)	2.12 (0.83–5.39)	2.21 (0.65–7.46)	2.59 (0.72–9.33)
Monocytes ≥ 0.55 × 10^3^/mL	0.77 (0.32–1.86)	0.83 (0.33–2.08)	0.75 (0.24–2.41)	1.02 (0.30–3.48)
Glucose > 100 mg/dL	0.94 (0.38–2.31)	1.09 (0.43–2.76)	1.25 (0.38–4.05)	1.31 (0.37–4.61)
Triglycerides ≥ 98 mg/dL	0.17 (0.05–0.57)	0.19 (0.05–0.67)	0.16 (0.04–0.61)	0.19 (0.05–0.79)
Cholesterol ≥ 189 mg/dL	0.59 (0.21–1.69)	0.50 (0.17–1.48)	0.69 (0.19–2.43)	0.51 (0.14–1.93)
HDL-cholesterol ≥ 56 mg/dL	2.53 (0.87–7.30)	1.46 (0.49–4.34)	1.88 (0.57–6.23)	1.12 (0.31–4.05)
PCR ≥ 1.5	1.93 (0.72–5.15)	1.28 (0.46–3.57)	2.41 (0.62–9.41)	1.41 (0.33–5.93)
NLR ≥ 3.3	0.68 (0.28–1.65)	0.61 (0.24–1.53)	1.00 (0.30–3.26)	0.92 (0.26–3.24)
PLR ≥ 142	0.36 (0.15–0.91)	0.41 (0.16–1.06)	0.28 (0.08–0.96)	0.35 (0.10–1.28)
LMR ≥ 3.0	2.63 (1.06–6.51)	2.12 (0.83–5.39)	1.56 (0.48–5.04)	1.21 (0.35–4.19)
SII ≥ 715	0.74 (0.30–1.79)	0.55 (0.22–1.39)	0.67 (0.20–2.21)	0.56 (0.16–2.00.16.00)
SIM ≥ 1.62	0.63 (0.26–1.52)	0.74 (0.29–1.85)	0.92 (0.29–2.99)	1.10 (0.31–3.82)

^a^Estimated from multinomial unconditional logistic regression model. ^b^Adjusted for sex, smoking, dizziness, time to treatment, and triglycerides level

## Discussion

This study investigated clinical and hematological predictors of hearing recovery in patients with ISSNHL treated with a combination of systemic and intratympanic corticosteroids. The aim was to explore whether easily accessible inflammatory blood markers could help identify individuals at greater risk of poor treatment response. Several factors—including presence of dizziness, smoking status, treatment delay, elevated triglycerides, and increased PLR—were independently associated with a reduced likelihood of hearing improvement.

All patients were treated with a combination of systemic and intratympanic corticosteroids, in line with evidence suggesting that dual-route therapy may enhance treatment efficacy compared to either modality alone [11–14]. This approach was adopted to maximize anti-inflammatory effects both systemically and locally at the cochlear level [15]: systemic corticosteroids ensure broad immunosuppressive action and vascular modulation, while intratympanic administration provides high drug concentrations in the inner ear compartment, potentially augmenting the therapeutic response.

Recently, the rationale and effectiveness of corticosteroid therapy in ISSNHL have been re-examined in major international studies. The HODOKORT randomized trial [16] found that high-dose systemic corticosteroids were not superior to standard-dose regimens for hearing recovery, while adverse events were more frequent in high-dose groups. The International Consensus (ICON) likewise recognizes systemic corticosteroids as the current standard of care, despite the limited and heterogeneous quality of evidence [17]. It recommends focusing future studies on moderate-to-profound hearing loss and supports intratympanic steroids mainly as a salvage option. Together, these works highlight the persistent uncertainty regarding optimal corticosteroid regimens and the need for individualized, evidence-based treatment strategies.

Despite this aggressive therapeutic approach, nearly one-third of patients in our series did not experience any hearing recovery, highlighting the complexity of ISSNHL pathophysiology and the need for better prognostic tools and treatment strategies. Among clinical variables, the presence of dizziness at onset was strongly associated with poor outcome. This finding is consistent with the view that vestibular involvement may indicate more extensive inner ear damage, potentially limiting the regenerative capacity of auditory structures [18, 19]. Smoking was another independent negative prognostic factor. Beyond its known systemic vascular and inflammatory effects, smoking may impair microcirculation in the cochlea, reduce oxygen delivery, and compromise corticosteroid efficacy [20]. Our findings suggest that smoking may negatively influence hearing recovery in ISSNHL, and support the consideration of smoking cessation as a potentially beneficial component of patient counseling and management, especially in individuals at higher risk of poor outcome.

Early initiation of treatment was associated with better outcomes, consistent with accumulating evidence. Corticosteroid therapy appears to be most effective when started within the first days after symptom onset, presumably because it can attenuate the inflammatory cascade before irreversible cochlear damage occurs. Our findings align with those of Chen et al. [21], who, in a large retrospective cohort of 666 patients, identified a clear temporal threshold at 14 days beyond which the efficacy of corticosteroids significantly declined. Their results demonstrated that hearing recovery was significantly greater in patients who initiated treatment within two weeks, with a marked reduction in improvement for those treated later. Notably, this effect was observed regardless of the severity of hearing loss and remained significant even after adjustment for potential confounders. Even within the framework of combined oral and intratympanic therapy, as adopted in our cohort, delayed treatment emerged as a strong predictor of therapeutic failure. These findings reinforce the importance of early clinical recognition and immediate intervention to maximize the chances of auditory recovery.

On a laboratory level, elevated triglycerides were associated with a worse prognosis. Dyslipidemia has been implicated in cochlear microangiopathy and may exacerbate endothelial dysfunction, promoting a pro-thrombotic environment [22–24]. Although total cholesterol levels were also higher in non-responders, triglycerides appeared to be a more robust marker in our analysis. Inflammatory indices derived from the complete blood count were also significant predictors. An elevated PLR was independently associated with poor hearing recovery, suggesting an underlying systemic inflammatory and pro-thrombotic state. High PLR has previously been identified as a negative prognostic indicator in several cardiovascular [25] and oncologic conditions [26]. In the setting of ISSNHL, it may reflect an activated platelet response combined with relative lymphopenia, favoring microvascular dysfunction within the cochlea. Notably, PLR retained predictive value even in patients receiving maximal anti-inflammatory treatment, indicating that this parameter may capture aspects of the host’s inflammatory profile that are not fully modifiable by corticosteroids. Conversely, a higher LMR was associated with a trend toward better recovery. LMR is thought to reflect the balance between adaptive immunity (lymphocytes) and innate, pro-inflammatory responses (monocytes). Although it did not reach independent significance in multivariable analysis, LMR may represent a secondary immunologic marker worth exploring in future studies.

Together, these findings suggest that both clinical characteristics and inflammatory markers can provide useful prognostic information even when patients are uniformly treated with intensive combination therapy. The identification of high-risk profiles may contribute to more informed clinical decision-making and patient counseling. While our data do not support specific therapeutic modifications, such profiles could, in the future, help guide the selection of patients who might benefit from adjunctive approaches. Interventions such as antiplatelet agents or hyperbaric oxygen therapy—whose efficacy remains under inconclusive—may be considered for evaluation in prospective trials targeting patients with unfavorable inflammatory or vascular markers [27].

This study has several strengths, including the homogeneity of treatment, rigorous selection criteria, and integration of clinical, metabolic, and immunologic data. Nevertheless, its retrospective design limits causal inference and is subject to selection bias. The absence of post-treatment hematological data prevents assessment of how inflammatory markers change in response to therapy. Additionally, all patients received combined corticosteroids, so we cannot evaluate the specific contribution of each route. However, the choice to standardize treatment enhances internal validity by reducing therapeutic variability.

In conclusion, even among patients treated with combined systemic and intratympanic corticosteroids, several clinical and hematologic parameters remain independently associated with hearing recovery. In particular, PLR appears to be a promising prognostic marker. Future prospective studies should validate these findings and explore the potential for integrating such markers into individualized treatment algorithms.

## Data Availability

The data supporting the results of this study are available from the corresponding author upon reasonable request.
